# Inhibition of Na_V_1.8 prevents atrial arrhythmogenesis in human and mice

**DOI:** 10.1007/s00395-020-0780-8

**Published:** 2020-02-20

**Authors:** Steffen Pabel, Shakil Ahmad, Petros Tirilomis, Thea Stehle, Julian Mustroph, Maria Knierim, Nataliya Dybkova, Philipp Bengel, Andreas Holzamer, Michael Hilker, Katrin Streckfuss-Bömeke, Gerd Hasenfuss, Lars S. Maier, Samuel Sossalla

**Affiliations:** 1 Department of Internal Medicine II, University Medical Center Regensburg, Franz-Josef-Strauß-Allee 11, 93053 Regensburg, Germany; 2 Clinic for Cardiology and Pneumology, Georg-August University Göttingen, Robert Koch Str. 40, 37075 Göttingen, Germany; 3 DZHK (German Centre for Cardiovascular Research), Partner Site Göttingen, Robert Koch Str. 40, 37075 Göttingen, Germany; 4 Department of Cardiothoracic Surgery, University Medical Center Regensburg, Franz-Josef-Strauß-Allee 11, 93053 Regensburg, Germany

**Keywords:** Antiarrhythmic drugs, Atrial arrhythmias, Na^+^ channel, Late sodium current

## Abstract

Pharmacologic approaches for the treatment of atrial arrhythmias are limited due to side effects and low efficacy. Thus, the identification of new antiarrhythmic targets is of clinical interest. Recent genome studies suggested an involvement of SCN10A sodium channels (Na_V_1.8) in atrial electrophysiology. This study investigated the role and involvement of Na_V_1.8 (SCN10A) in arrhythmia generation in the human atria and in mice lacking Na_V_1.8. Na_V_1.8 mRNA and protein were detected in human atrial myocardium at a significant higher level compared to ventricular myocardium. Expression of Na_V_1.8 and Na_V_1.5 did not differ between myocardium from patients with atrial fibrillation and sinus rhythm. To determine the electrophysiological role of Na_V_1.8, we investigated isolated human atrial cardiomyocytes from patients with sinus rhythm stimulated with isoproterenol. Inhibition of Na_V_1.8 by A-803467 or PF-01247324 showed no effects on the human atrial action potential. However, we found that Na_V_1.8 significantly contributes to late Na^+^ current and consequently to an increased proarrhythmogenic diastolic sarcoplasmic reticulum Ca^2+^ leak in human atrial cardiomyocytes. Selective pharmacological inhibition of Na_V_1.8 potently reduced late Na^+^ current, proarrhythmic diastolic Ca^2+^ release, delayed afterdepolarizations as well as spontaneous action potentials. These findings could be confirmed in murine atrial cardiomyocytes from wild-type mice and also compared to SCN10A^−/−^ mice (genetic ablation of Na_V_1.8). Pharmacological Na_V_1.8 inhibition showed no effects in SCN10A^−/−^ mice. Importantly, in vivo experiments in SCN10A^−/−^ mice showed that genetic ablation of Na_V_1.8 protects against atrial fibrillation induction. This study demonstrates that Na_V_1.8 is expressed in the murine and human atria and contributes to late Na^+^ current generation and cellular arrhythmogenesis. Blocking Na_V_1.8 selectively counteracts this pathomechanism and protects against atrial arrhythmias. Thus, our translational study reveals a new selective therapeutic target for treating atrial arrhythmias.

**Electronic supplementary material:**

The online version of this article (10.1007/s00395-020-0780-8) contains supplementary material, which is available to authorized users.

## Introduction

Atrial arrhythmias, in particular atrial fibrillation (AF), contribute to the morbidity and mortality of western societies [4]. However, pharmacological therapeutic options are still limited due to moderate potency and severe side effects. Therefore, identification and evaluation of new targets involved in atrial arrhythmogenesis are of clinical interest. The mechanisms for atrial arrhythmogenesis include electrical remodelling and disturbances in ion homeostasis [16]. Both can cause focal triggered activity, which might evoke atrial arrhythmias or promote re-entry mechanisms. One potent substrate in promoting electrical disturbances and focal triggered activity in the atria is an increased late Na^+^ current (*I*_NaL_), which is a persistent Na^+^ influx throughout the action potential [3, 16, 22, 33]. By prolonging the duration of the action potential, *I*_NaL_ increases the probability of early afterdepolarizations (EADs), which constitute a trigger for arrhythmias. Moreover, by increasing cytosolic [Na^+^] an enhanced *I*_NaL_ may lead to Na^+^/Ca^2+^ exchanger (NCX)-mediated Ca^2+^ overload [26]. Consecutively, this induces arrhythmogenic Ca^2+^ release events (Ca^2+^ sparks) from the sarcoplasmic reticulum (SR) during diastole [12, 13]. Increasing diastolic Ca^2+^ levels may promote a depolarizing inward current (*I*_ti_), resulting in delayed afterdepolarizations (DADs), which serve as a trigger for irregular action potentials and focal arrhythmias [32]. However, the mechanisms involved in *I*_NaL_ generation in the atria are not fully understood.

While SCN5A sodium channels (Na_V_1.5) are the predominant isoform in the heart [14], recent evidence suggested the involvement of SCN10A sodium channels (Na_V_1.8) in atrial conduction [8]. Moreover, genome-wide association studies showed that variants of Na_V_1.8 are associated with the development of atrial fibrillation [17, 21, 25]. Therefore, the aim of our study was to fundamentally investigate the molecular and functional role of Na_V_1.8 in the human and murine atria. Moreover, we studied the involvement of Na_V_1.8 in atrial arrhythmogenesis and evaluated the channel as a specific target for antiarrhythmic pharmacotherapy.

## Materials and methods

### Human myocardial tissue

All procedures were performed according to the Declaration of Helsinki and were approved by the local ethics committee. Informed consent was obtained from all patients. Human atrial myocardium from patients with sinus rhythm or atrial fibrillation was acquired from atrial resections during open heart surgery (for patient characteristics, see Table 1). For molecular purposes, we utilized left ventricular myocardium from healthy donor hearts that were not transplanted due to technical reasons.

**Table 1:** Clinical characteristics of patients with sinus rhythm (*n* = 34) and patients with atrial fibrillation (*n* = 10)

Patient data	Sinus rhythm (*n* = 34)	Atrial fibrillation (*n* = 10)	Non-failing ventricle (*n* = 10)^a^
Male sex (%)	73.5	50.0	N/A
Age (Mean ± SEM, y)	64.56 ± 1.55	75.2 ± 2.55	N/A
EF (Mean ± SEM, %)	56.89 ± 1.95	51.25 ± 3.03	N/A
Ischemic heart disease (%)	100.0	100.0	N/A
Diabetes (%)	41.4	20.0	N/A
ACE inhibitors (%)	83.3	80.0	N/A
β-Blockers (%)	81.8	100.0	N/A
Digoxin (%)	0.0	0.0	N/A
Catecholamines (%)	0.0	0.0	N/A
Amiodaron (%)	0.0	30.0	N/A
PDE inhibitors (%)	0.0	0.0	N/A
Statin (%)	83.9	90.0	N/A

Values are mean ± SEM or *n* (%). Clinical data could not be completely obtained from every patient

*EF* ejection fraction, *ACE* angiotensin-converting enzyme, *PDE* phosphodiesterase

^a^Blinded due to ethical reasons

### SCN10A^−/−^ and wild-type mice

SCN10A^−/−^ and respective wild-type mice (WT) were studied to reveal the functional consequence of genetic Na_V_1.8 ablation [2]. The animal investigations conform to the “Guide for the Care and Use of Laboratory Animals” published by the US National Institutes of Health (publication No. 85-23, revised 1996) and the guidelines from Directive 2010/63/EU of the European Parliament on the protection of animals used for scientific purposes. Murine atrial cardiomyocytes were isolated as previously reported [13]. Mice used for cardiomyocyte isolation were sacrificed under isoflurane 133 inhalation anesthesia (5%) by cervical dislocation. Mice used for in vivo studies were anesthetized via intraperitoneal injection of medetomidine (0.5 mg/kg), midazolam (5 mg/kg) and fentanyl (0.05 mg/kg body weight) and killed by cervical dislocation after the procedure.

### Human atrial cardiomyocytes isolation

Atrial myocardium from patients with sinus rhythm was used for cellular experiments. Before starting isolation, human atrial tissue was cleared from fat and blood, then cut into very small pieces and rinsed thoroughly. Cardiomyocytes were isolated using collagenase (Worthington type 1, 370 U/mg) and proteinase (Sigma Type XXIV, 7.0–14.0 U/mg) as described previously [15]. Enzymatic digestion was stopped by adding BCS (2%). The supernatant containing dispersed cells was centrifuged (58 g, 5 min) and cells were resuspended in storage medium. Only cell solutions containing elongated cardiomyocytes with clear cross-striations were selected for experiments, plated on laminin-coated recording chambers, and left to settle for 30 min. A representative isolated human atrial cardiomyocyte is shown in Fig. 2a. Cellular experiments were performed at room temperature.

### Murine atrial cardiomyocytes isolation

Atrial cardiomyocytes from SCN10A^−/−^ and respective WT mouse hearts were isolated as previously described [13]. Cellular experiments were performed at room temperature.

### Quantitative real-time PCR (qPCR)

Human atrial tissue or ventricular non-failing tissue were snap-frozen in liquid nitrogen and stored at − 80 °C. RNA was isolated by use of the SV total RNA isolation System (Promega). Primer sequences (forward and four reverse) of SCN10A (Origene, cat No HP209444), SCN5A and GAPDH were used for quantitative RT-PCR.

### Western blots

Human atrial tissue samples from patients with sinus rhythm (SR) and atrial fibrillation (AF) as well as human ventricular samples from healthy donors (NF) were homogenized in Tris buffer and complete protease and phosphatase inhibitor cocktails (Roche Diagnostics). Protein concentration was determined by BCA assay (Pierce Biotechnology). Mouse monoclonal anti-Na_V_1.8 antibodies (1:1,000, LSBio, LS-C109037), rabbit polyclonal anti-Na_V_1.5 (1:2,000, Alomone labs, ASC-005), and mouse monoclonal anti-GAPDH (1:20,000, BIOTREND, BTMC-A473-9) were used. ImmobilonTM Western Chemiluminescent HRP Substrate (Millipore) was used for the chemiluminescent detection.

### Pharmacological interventions

For selectively blocking Na_V_1.8, isolated cardiomyocytes were treated with either A-803467 (30 nmol/L, Sigma) or PF-01247324 (1 µmol/L, Sigma). Cells were incubated for 15 min before measurements were started. Isoproterenol (30 nmol/L, Sigma) was used for slight beta-adrenergic stimulation in all groups [10]. Moreover, we used tetrodotoxin (2 µmol/l) to inhibit *I*_NaL_.

### Patch-clamp experiments

#### ***I***_NaL_ measurements

Ruptured-patch whole-cell voltage-clamp was used to measure *I*_NaL_ in human atrial cardiomyocytes (HEKA electronics). Cardiomyocytes were held at − 120 mV and *I*_NaL_ was elicited using a train of pulses to − 35 mV (1 s duration, ten pulses, BCL 2 s). Recordings were initiated 3 min after rupture. The measured current was integrated (between 100 and 500 ms) and normalized to the membrane capacitance (Suppl. Fig. 3).

#### Action potential recordings

For action potential recordings, the whole-cell patch-clamp technique was used (current clamp configuration, HEKA electronics). Access resistance was typically ~ 5–10 MΩ after patch rupture. Action potentials were continuously elicited by square current pulses of 0.5–1 nA amplitude and 1–5 ms duration at a frequency of 1 Hz. For assessing DADs and spontaneous action potentials, stimulation was paused for 15 s and for 30 s.

### Confocal Ca^2+^ spark measurements

Isolated atrial cardiomyocytes were loaded with the Ca^2+^ indicator Fluo 4-AM (10 µmol/L for 15 min, Molecular Probes) at RT. The solution was substituted and cells were incubated for 15 min with Tyrode’s solution and the respective agents. Line scans for Ca^2+^ spark measurements were obtained with a laser scanning confocal microscope (Zeiss). Line scans were recorded during rest after loading the sarcoplasmic reticulum with Ca^2+^ by continuous field stimulation at 1 Hz. Ca^2+^ sparks were analysed with the program SparkMaster for ImageJ.

### In vivo arrhythmia studies

For electrophysiological studies, SCN10A^−/−^ and respective wild-type mice [2] were anesthetized and temperature controlled (37 °C). As previously described, a Millar 1.1F octapolar EP catheter (EPR-800; Millar Instruments) was inserted via the right jugular vein [20]. Right atrial pacing was performed using 2 ms current pulses delivered by an external stimulator. Atrial capture was confirmed by atrial pacing prior to the arrhythmia protocol. Inducibility of atrial arrhythmias was tested by decremental burst pacing (5 episodes/mice). AF was defined as the occurrence of rapid and fragmented atrial electrograms with irregular AV nodal conduction and ventricular rhythm for at least 1 s.

### Statistics

All data are presented as the mean values ± SEM. For statistical analysis of two groups containing parametric data Student’s *t* test was used, for non-parametric data Mann–Whitney test was used.

For analysis of parametric data comparing more than two groups, one-way ANOVA was used. *P* values were corrected for multiple comparisons by the Tukey method. For analysis of proportions, Fisher's exact test was used. Analysis was performed using GraphPad Prism 8. *P* values are two-sided and considered statistically significant if *P* < 0.05.

## Results

### Expression of Na_V_1.8 in human atrial myocardium

**Fig. 1:**
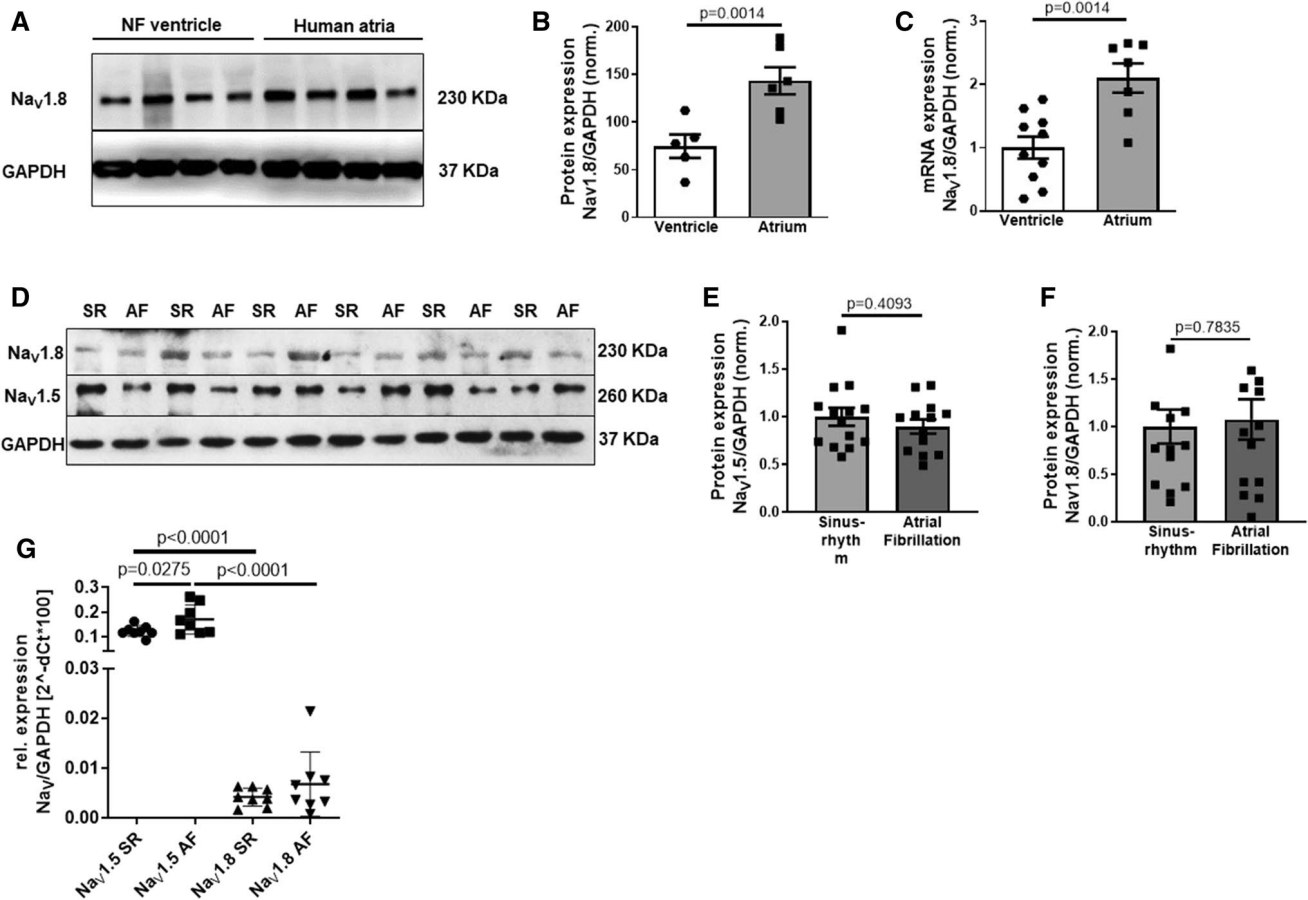
Expression of Na_V_1.8 in the human atrium. Data are presented as scatter plot with mean ± SEM. *P* values were calculated using unpaired Student's *t* test. **a** Original Western blot for Na_V_1.8 expression in atrial myocardium from patients with sinus rhythm compared to ventricular myocardium from non-failing donors (NF). **b** Normalized densitometry data comparing the protein expression of Na_V_1.8 in human atria (*n* = 6) and human ventricle (*n* = 5). GAPDH was used as an internal loading control in all blots. **c** Normalized mRNA expression of Na_V_1.8/GAPDH in human atrial myocardium (*n* = 7 patients) compared to ventricular myocardium from healthy subjects (*n* = 10 patients). **d** Original Western Blot for Na_V_1.8 and Na_V_1.5 protein in human atria from patients with sinus rhythm (SR) or atrial fibrillation (AF). **e** Normalized densitometry data from Western Blots using atrial myocardium from patients with SR or AF showing the protein expression of Na_V_1.5 (SR: *n* = 14 patients, AF: *n* = 13) and (**f**) Na_V_1.8 (SR: *n* = 14 patients, AF: *n* = 14). GAPDH was used as an internal loading control in all blots. **g** Normalized mRNA expression of Na_V_1.5/GAPDH (SR: *n* = 8 patients, AF: *n* = 8) and Na_V_1.5/GAPDH (SR: *n* = 8 patients, AF: *n* = 8) in human atrial myocardium from SR compared to AF

To investigate whether Na_V_1.8 is expressed in the human atrium, we used myocardium from patients with SR and patients with AF for mRNA and protein analysis. At the protein level, we could confirm the existence of Na_V_1.8 in the human atria. Moreover, Na_V_1.8 protein expression is significantly higher in the human atria (*n* = 6) compared to the human ventricle (*n* = 5 Fig. 1a, b). Using qPCR, we detected the expression of Na_V_1.8 mRNA in human atrial tissue, which was 3.0 ± 0.9-fold higher in the human atrium as compared to ventricular non-failing myocardium (ventricle: *n* = 10 patients, atria: *n* = 7 patients, Fig. 1c). To evaluate whether Na_V_1.8 or the major cardiac sodium channel isoform Na_V_1.5 might be differentially regulated in atrial fibrillation (AF) compared to sinus rhythm (SR), we investigated atrial myocardium from patients with SR or with AF. However, neither Na_V_1.8 (SR: *n* = 14 patients, AF: *n* = 14 patients) nor Na_V_1.5 (SR: *n* = 14 patients, AF: *n* = 13 patients) protein expression levels were different between myocardium from patients with AF and SR (Fig. 1d–f). Moreover, while Na_V_1.8 mRNA was lower compared to Na_V_1.5, we found no changes between SR versus AF for Na_V_1.8 (SR: *n* = 8 patients, AF: *n* = 8). Na_V_1.5 mRNA levels differed between SR (*n* = 8 patients) and AF (*n* = 8, Fig. 1g), which however did not translate into protein expression differences. Therefore, Na_V_1.8 was confirmed to be present in the human atria without being regulated in patients with AF.

### Effects of Na_V_1.8 on the cardiac action potential

**Fig. 2:**
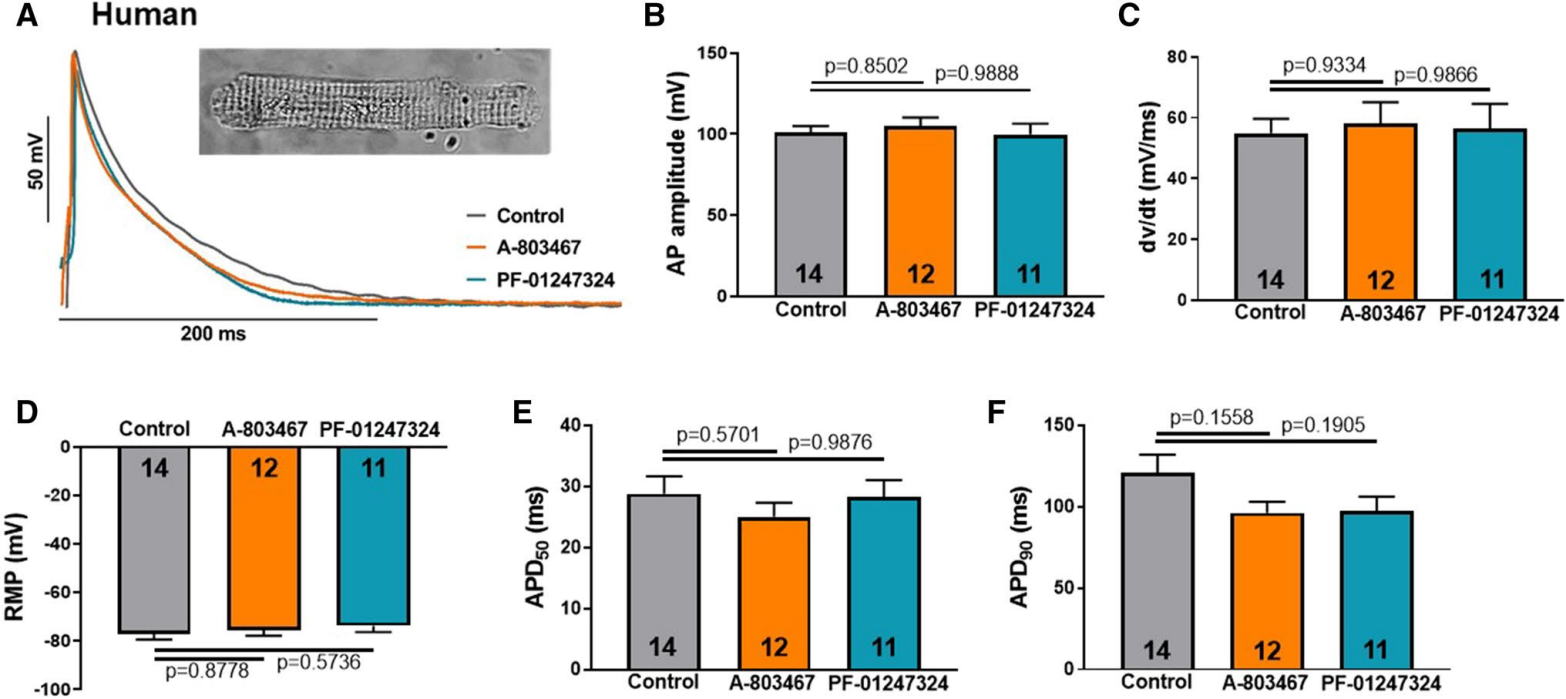
Effects of Na_V_1.8 on the human atrial action potential. Data are presented as mean ± SEM. *P* values were computed using one-way ANOVA with Tukey's test for multiple comparisons. **a** Representative action potential recordings (1 Hz stimulation). Inset: isolated human atrial cardiomyocyte. **b** Effects of Na_V_1.8 inhibition by A-803467 (*n* = 12 cardiomyocytes/5 patients) or PF-01247324 (*n* = 11/5) compared to control (*n* = 14/5) on action potential (AP) amplitude, (**c**) maximum upstroke velocity (*dv*/*dt*), (**d**) resting membrane potential (RMP) and action potential duration at (**e**) 50% (APD_50_) and (**f**) 90% repolarization (APD_90_)

**Fig. 3:**
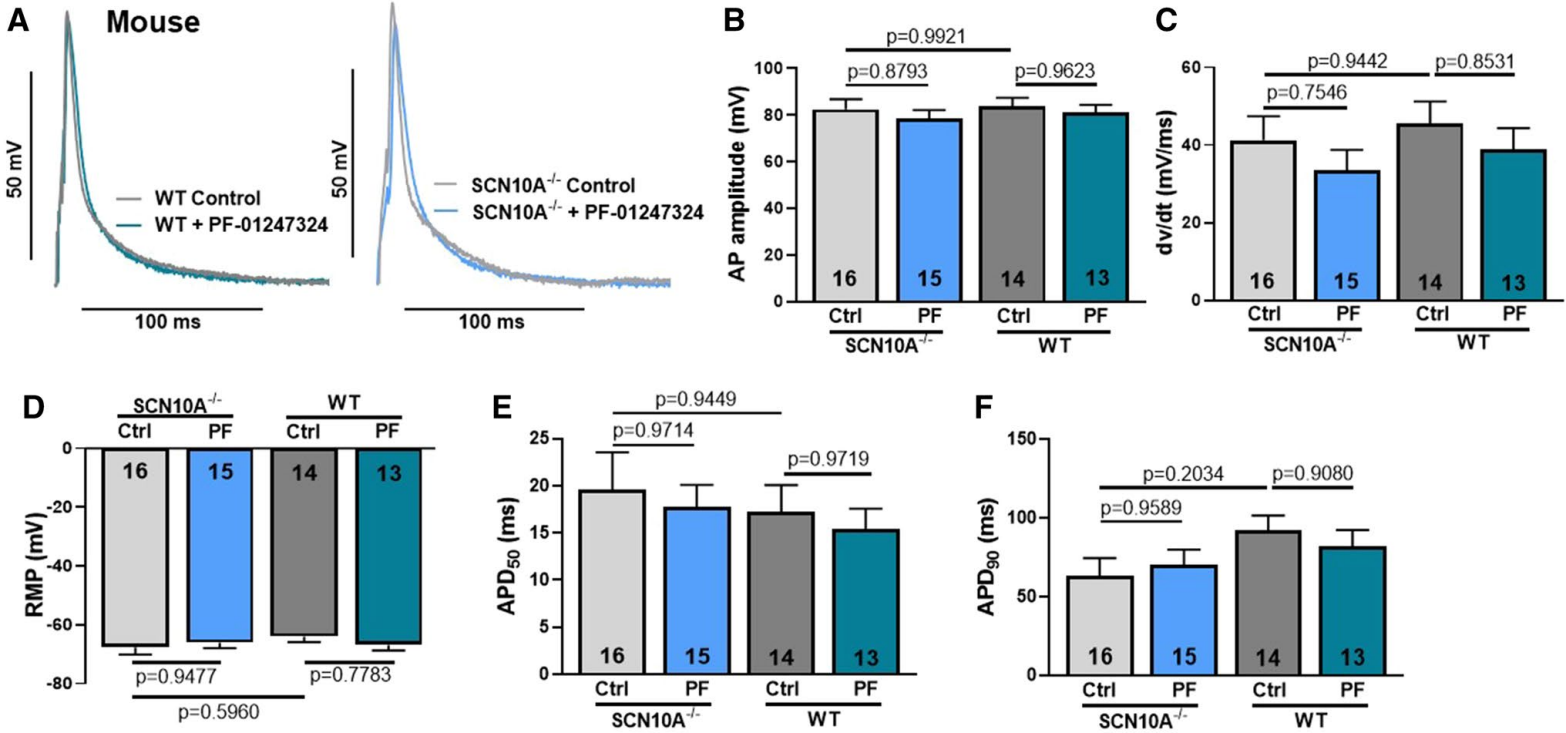
Effects of Na_V_1.8 on the murine atrial action potential using SCN10A^−/−^ and WT mice. Data are presented as mean ± SEM. *P* values were calculated using one-way ANOVA with Tukey's test for multiple comparisons. **a** Representative action potential recordings (1 Hz stimulation) of isolated murine atrial cardiomyocytes. **b** Effects of genetic ablation of Na_V_1.8 (SCN10A^−/−^: *n* = 16 cells/10 mice) compared to WT (*n* = 14 cells/8 mice) and effects of pharmacological inhibition of Na_V_1.8 by PF-01247324 in each genotype (SCN10A^−/−^: *n* = 15 cells/10 mice and WT: *n* = 13/8) on action potential (AP) amplitude, (**c**) maximum upstroke velocity (*dv*/*dt*), (**d**) resting membrane potential (RMP) and action potential duration at (**e**) 50% (APD_50_) and (**f**) 90% repolarization (APD_90_)

To investigate the effect of Na_V_1.8 on the action potential properties in human atrial cardiomyocytes, we performed ruptured-patch whole-cell current clamp experiments using freshly isolated cardiomyocytes from patients with sinus rhythm (Fig. 2a). Action potential amplitude (APA), maximum upstroke velocity (*dv*/*dt*), action potential duration (APD) as well as resting membrane potential (RMP) were investigated from five different patients with sinus rhythm (control: *n* = 14 cardiomyocytes; A-803467: *n* = 12; PF-01247324: *n* = 11). APA (100.9 ± 4.1 mV) and *dv*/*dt* (54.88 ± 4.8 mV/ms) were not altered after inhibition of Na_V_1.8 by A-803467 (APA: 104.9 ± 5.5 mV; *dv*/*dt*: 58.08 ± 7.0 mV/ms) or PF-01247324 (APA: 99.8 ± 6.7 mV; *dv*/*dt*: 56.33 ± 8.2 mV/ms), which indicates that Na_V_1.8 has negligible effects on peak Na^+^ current (Fig. 2b, c). Also, RMP (-77.1 ± 2.3 mV) was not changed after Na_V_1.8 inhibition (A-803467: − 75.6 ± 2.3 mV; PF-01247324: − 73.9 ± 2.5 mV, Fig. 2d). APD at 50% repolarization (APD_50_) was 28.8 ± 2.6 ms in control compared to A-803467 (25.0 ± 2.3 ms) and PF-01247324 (28.2 ± 2.8 ms, Fig. 2e). However, APD_90_ was slightly abbreviated (APD_90_; 121.0 ± 11.0 ms) after exposure to A-803467 (96.3 ± 6.9 ms) and PF-01247324 (97.2 ± 9.1 ms, Fig. 2f), which, however, did not reach statistical significance. We further evaluated the effects of Na_V_1.8 on the action potential in atrial cardiomyocytes from SCN10A^−/−^ (*n* = 10 mice, control: *n* = 16 cells, PF-01247324: *n* = 15 cells) and WT mice (*n* = 8 mice, control: *n* = 14 cells, PF-01247324: *n* = 13 cells, Fig. 3a). According to the human data, we could confirm that Na_V_1.8 has no effects on APA (Fig. 3b), *dv/dt* (Fig. 3c), RMP (Fig. 3d) as well as APD_50_ (Fig. 3e) or APD_90_ (Fig. 3f). Accordingly, we observed no effects of pharmacological Na_V_1.8 inhibition using PF-01247324 in SCN10A^−/−^ and WT. These experiments indicate that Na_V_1.8 has negligible effects on the human and murine atrial action potential, which is of importance for further translational studies.

### Role of Na_V_1.8 for generation of ***I***_NaL_

**Fig. 4:**
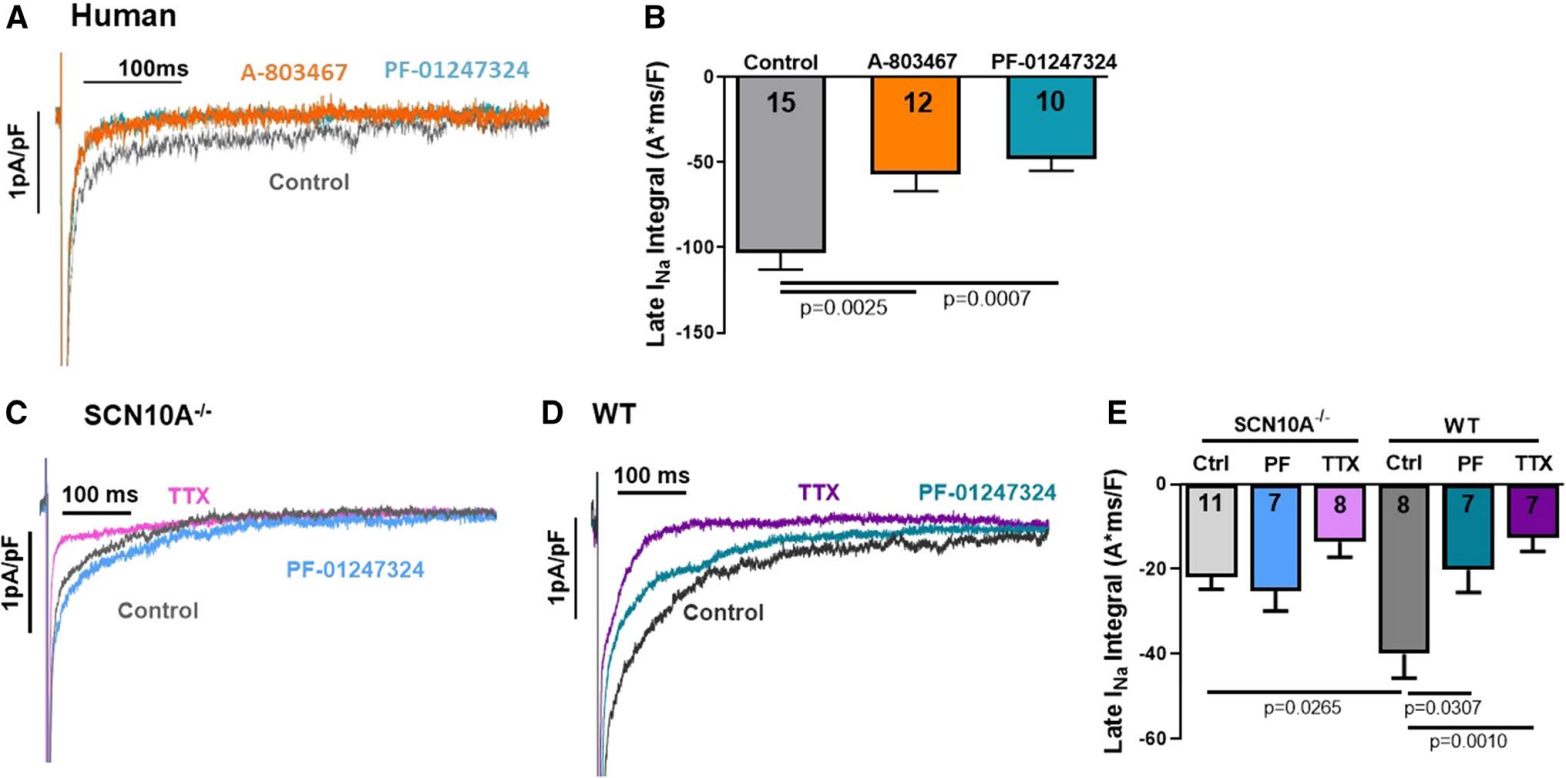
Role of Na_V_1.8 in *I*_NaL_ generation. Values are reported as mean ± SEM. One-way ANOVA with Tukey's test for multiple comparisons was used to calculate *P* values. **a** Original traces of *I*_NaL_ in human atrial cardiomyocytes and (**b**) mean data of *I*_NaL_ (integral 100–500 ms) showing the effects of Na_V_1.8 inhibition with either A-803467 (*n* = 12 cardiomyocytes/4 patients) or PF-01247324 (*n* = 10/4) on *I*_NaL_ compared to control (*n* = 15/6). **c** Original *I*_NaL_ traces representing *I*_NaL_ in SCN10A^−/−^ mice as well as (**d**) WT and *I*_NaL_ after Na_V_1.8 inhibition by PF-01247324 and tetrodotoxin treatment respectively. **e** Mean values of *I*_NaL_ (integral 100–500 ms) showing the effects of genetic ablation of Na_V_1.8 (SCN10A^−/−^: *n* = 11 cells/5 mice) compared to WT (*n* = 8 cells/4 mice) and effects of pharmacological inhibition of Na_V_1.8 by PF-01247324 (SCN10A^−/−^: *n* = 7 cells/5 mice and WT: *n* = 7/4) and TTX (SCN10A^−/−^: *n* = 8 cells/5 mice and WT: *n* = 7/4) in each genotype

The contribution of Na_V_1.8 in *I*_NaL_ generation was studied in human atrial cardiomyocytes from patients with sinus rhythm using ruptured-patch whole-cell voltage-clamp. Patch-clamp recordings of isolated human atrial cardiomyocytes showed that Na_V_1.8 inhibition caused a significant reduction of *I*_NaL_ by 44.9 ± 13.5% after exposure to A-803467 (*n* = 12 cardiomyocytes/4 patients) and by 53.5 ± 12.7% after PF-01247324 (*n* = 10/4) compared to control (*n* = 15/6, Fig. 4a, b). Furthermore, isolated atrial cardiomyocytes from SCN10A^−/−^ mice (*n* = 11 cells/ 5 mice) showed a significantly lower *I*_NaL_ compared to WT (*n* = 8 cells/ 4 mice, Fig. 4c–e). While pharmacological Na_V_1.8 inhibition by PF-01247324 (*n* = 7 cells/ 5 mice) exerted no effect on *I*_NaL_ in SCN10A^−/−^, *I*_NaL_ could be significantly reduced by application of PF-01247324 in atrial WT cardiomyocytes (*n* = 7 cells/ 4 mice, Fig. 4c–e). To determine the contribution of Na_V_1.8 to *I*_NaL_ generation, we also performed measurements with TTX (2 µmol/L) to globally inhibit *I*_NaL_. We observed a trend towards a lower *I*_NaL_ in TTX-treated SCN10A^−/−^ cardiomyocytes and also compared to PF-01247324-treated WT cardiomyocytes (Fig. 4e) suggesting that other Na_V_-dependent *I*_NaL_ is still relevant under these conditions.

### Role of Na_V_1.8 for sarcoplasmic Ca^2+^-leak generation

**Fig. 5:**
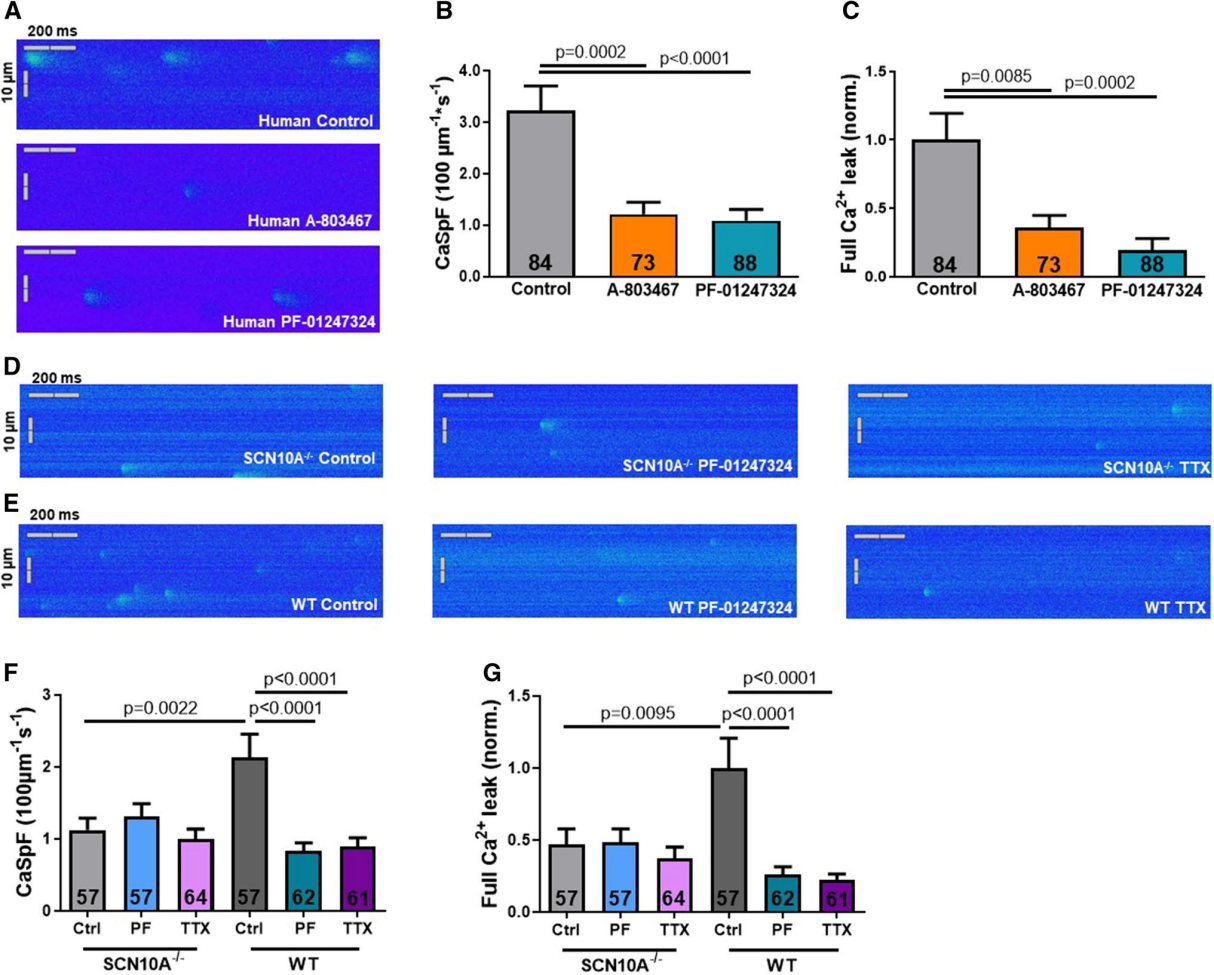
Relevance of Na_V_1.8 for diastolic sarcoplasmic Ca^2+^ leak. Values are given as mean ± SEM. One-way ANOVA with Tukey's test for multiple comparisons was used to calculate *P* values. **a** Representative confocal line scan images of human atrial cardiomyocytes loaded with the Ca^2+^ indicator Fluo-4 showing Ca^2+^ sparks during diastole. **b** Mean values of the frequency of Ca^2+^ sparks (CaSpF) and (**c**) the total calculated diastolic Ca^2+^ leak in atrial cardiomyocytes (*n* = 84 cardiomyocytes/13 patients) and after blocking Na_V_1.8 with A-803467 (*n* = 73/9) or PF-01247324 (*n* = 88/11). **d** Original line scan images of murine atrial cardiomyocytes from SCN10A^−/−^ and (**e**) WT mice. **f** Mean values of CaSpF and (**g**) the total calculated diastolic Ca^2+^ leak in SCN10A^−/−^ (*n* = 57 cells/7 mice) and WT (*n* = 57 cells/7 mice) mice and, respectively, effects of Na_V_1.8 inhibition by PF-01247324 (SCN10A^−/−^: *n* = 57 cells/7 mice and WT: *n* = 62/7) and TTX (SCN10A^−/−^: *n* = 64 cells/7 mice and WT: *n* = 61/7)

It is well known that *I*_NaL_ can potently induce arrhythmogenic diastolic SR Ca^2+^ release events [13]. To investigate whether the reduction of *I*_NaL_ caused by Na_V_1.8 inhibition may lead to a diminished incidence of diastolic SR Ca^2+^ release in human atrial cardiomyocytes we used confocal microscopy. The frequency of diastolic SR Ca^2+^ sparks (CaSpF) in line scans of human atrial cardiomyocytes was 3.2 ± 0.5 × 100/µm/s (*n* = 84 cardiomyocytes/13 patients) which could be significantly attenuated to 1.2 ± 0.2 × 100/µm/s after addition of A-803467 (*n* = 73/9) or PF-01247324 to 1.1 ± 0.2 × 100/µm/s (*n* = 88/11, Fig. 5a, b). Moreover, the calculated diastolic SR Ca^2+^ leak was reduced by 64.3 ± 22.2% after addition of A-803467 and by 80.6 ± 20.6% after PF-01247324 (control: *n* = 84 cardiomyocytes/13 patients, A-803467: *n* = 73/9; PF-01247324: *n* = 88/11, Fig. 5c). In line with that, atrial cardiomyocytes from SCN10A^−/−^ mice (*n* = 57 cells/ 7 mice, Fig. 5d) showed a lower frequency of diastolic Ca^2+^ sparks as well as a lower diastolic Ca^2+^ leak compared to WT cells (*n* = 57 cells/ 7 mice, Fig. 5e–g). While PF-01247324- (*n* = 62 cells/7 mice) and TTX- (*n* = 61 cells/ 7 mice) treated cardiomyocytes had a significantly reduced Ca^2+^ spark frequency and Ca^2+^ leak in WT, both drugs had no further effects in SCN10A^−/−^ cardiomyocytes (*n* = 57 cells/7 mice and 64/7 respectively). Interestingly, we observed no further antiarrhythmic effect of TTX compared to Na_V_1.8 inhibition alone. Thus, the reduction of both *I*_NaL_ and diastolic SR Ca^2+^ release reveal the significant role of Na_V_1.8 for cellular arrhythmogenesis in the human atria. Of note, we observed no effects of pharmacological Na_V_1.8 inhibition on systolic Ca^2+^ transient amplitude and SR Ca^2+^ load (Suppl. Figs. 1,  2).

### Blocking Na_V_1.8 suppresses diastolic Ca^2+^ waves

**Fig. 6:**
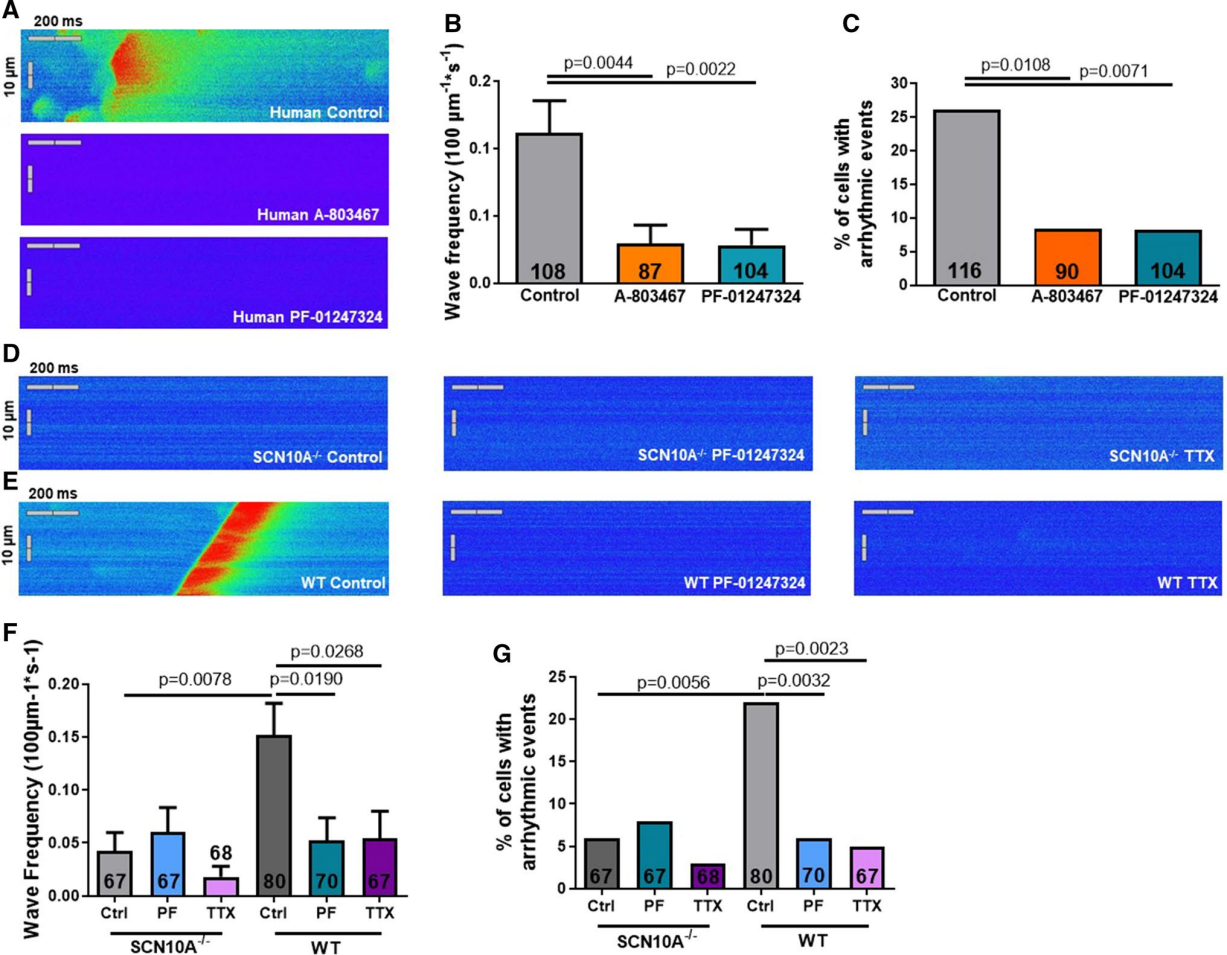
Effects of Na_V_1.8 on diastolic Ca^2+^ waves. Data are presented as mean ± SEM. *P* values were calculated using one-way ANOVA with Tukey's test for multiple comparisons or Fisher's exact test (for **c**, **g**). **a** Confocal line scan images of human atrial cardiomyocytes loaded with the Ca^2+^ indicator Fluo-4 representing the occurrence of major diastolic arrhythmogenic Ca^2+^ release events. **b** Mean frequency of Ca^2+^ waves in control (*n* = 108 cardiomyocytes/13 patients) and after treatment with A-803467 (*n* = 87/9) or PF-01247324 (*n* = 104/11). **c** Percentage of cells developing arrhythmic events (Ca^2+^ waves or spontaneous transients; *n* = 24 of 116 cardiomyocytes/13 patients) and effects of Na_V_1.8 blockade with A-803467 (*n* = 7 of 90/9 patients) or PF-01247324 (*n* = 8 of 104/11 patients). **d** Representative original line scans of murine atrial cardiomyocytes from SCN10A^−/−^ and (**e**) WT mice. **f** Mean values of Ca^2+^ wave frequency and (**g**) proportion of cells showing arrhythmic events in SCN10A^−/−^ (*n* = 67 cells/7 mice) and WT (*n* = 80 cells/7 mice) mice and effects of Na_V_1.8 inhibition by PF-01247324 (SCN10A^−/−^: *n* = 67 cells/7 mice and WT: *n* = 70/7) and TTX (SCN10A^−/−^: *n* = 68 cells/7 mice and WT: *n* = 67/7) in both genotypes

To elucidate potential antiarrhythmic effects of Na_V_1.8 inhibition, we have studied the effects of pharmacological inhibition and genetic ablation of Na_V_1.8 on the incidence of diastolic Ca^2+^ waves, which are major diastolic Ca^2+^ release events and constitute proarrhythmic triggers. Indeed, human atrial cardiomyocytes from patients with sinus rhythm treated with A-803467 or PF-01247324 showed a significantly reduced frequency of diastolic Ca^2+^ waves (Fig. 6a, b). Also, the percentage of cells developing diastolic Ca^2+^ waves, 26.1% under control conditions (*n* = 116 cardiomyocytes/13 patients), decreased after exposure to either A-803467 (to 8.4%, *n* = 90/9) or PF-01247324 (to 8.3%, *n* = 104/11, Fig. 6c). Also, SCN10A^−/−^ mice (*n* = 67 cells/7 mice) had a reduced Ca^2+^ wave frequency as well as a reduced fraction of cardiomyocytes with arrhythmic events compared to WT (*n* = 80 cells/7 mice, Fig. 6d–g). Pharmacological Na_V_1.8 inhibition with PF-01247324 exerted no effects in SCN10A^−/−^ mice (*n* = 67 cells/7 mice), but significantly decreased Ca^2+^ wave frequency and the percentage of cells with arrhythmic events in WT (*n* = 70 cells/7 mice, Fig. 6f–g). *I*_NaL_ inhibition by TTX caused no further effects compared to Na_V_1.8 ablation (*n* = 68 cells/7 mice) or inhibition (*n* = 67 cells/7 mice Fig. 6f–g).

### Inhibition of Na_V_1.8 reduces proarrhythmic triggers in atrial cardiomyocytes

**Fig. 7:**
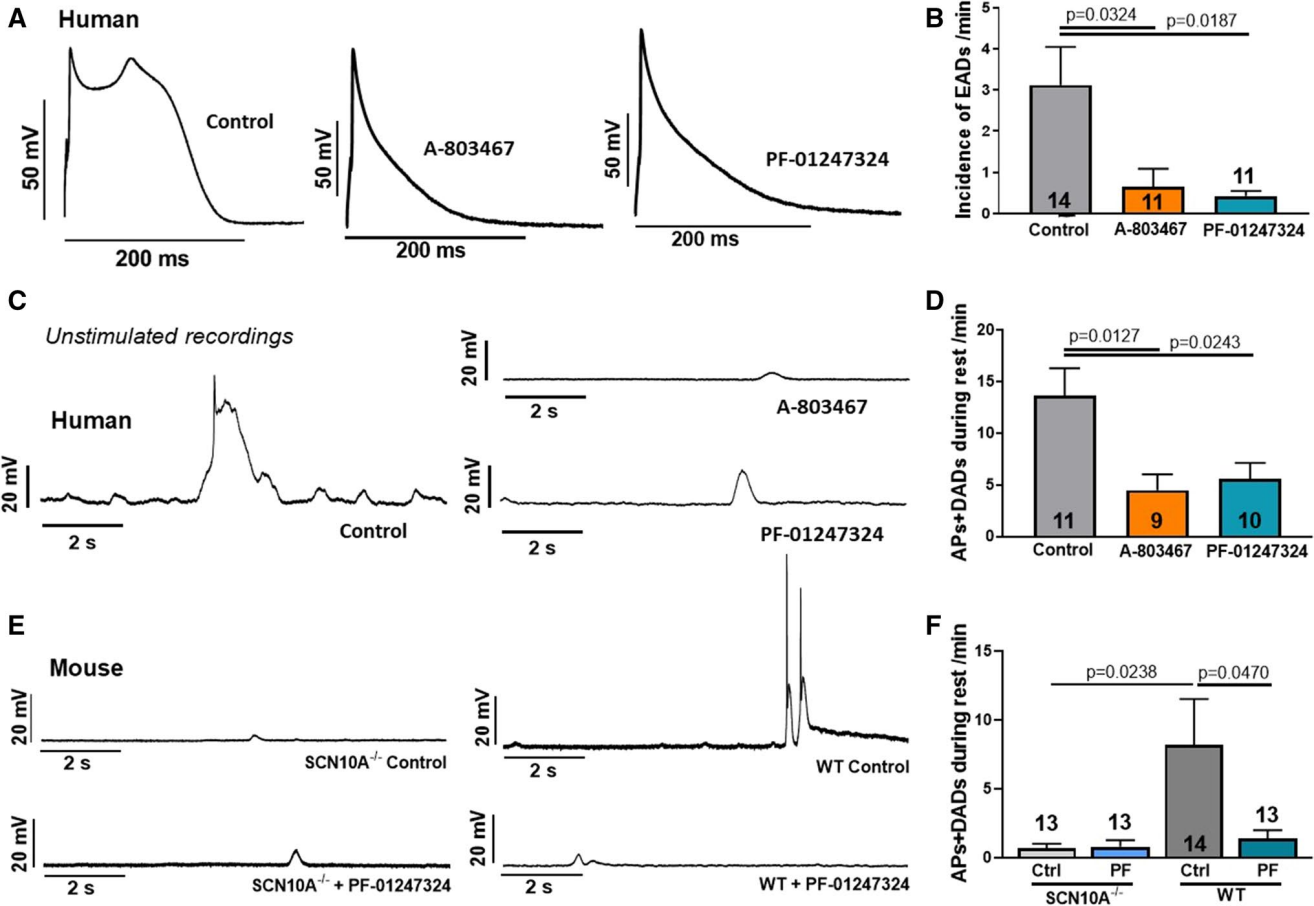
Effects of Na_V_1.8 inhibition on cellular arrhythmogenic trigger. Data are reported as mean ± SEM. *P* values were calculated using one-way ANOVA with Tukey's test for multiple comparisons. **a** Action potential recordings (1 Hz) representing the occurrence of an early afterdepolarization (EAD). **b** Incidence of EADs/min (*n* = 14 cardiomyocytes/4 patients) and effects of inhibiting Na_V_1.8 with A-803467 (*n* = 11/4) or PF-01247324 (*n* = 11/4). **c** Original unstimulated recordings of human atrial cardiomyocytes during 10 s rest after a series of 30 stimulated action potentials (1 Hz). **d** Mean incidence of spontaneous action potentials (APs) and delayed afterdepolarizations (DADs) during rest (*n* = 11/4) and after treatment with A-803467 (*n* = 9/4) or PF-01247324 (*n* = 10/4). **e** Original unstimulated recordings of murine atrial cardiomyocytes during 10 s rest after a series of 30 stimulated action potentials (1 Hz) from SCN10A^−/−^ and WT mice. **f** Mean values of spontaneous APs and DADs during rest in SCN10A^−/−^ (*n* = 13 cells/ 10 mice) and WT (*n* = 14 cells/ 8 mice) and effects of PF-01247324 in SCN10A^−/−^ (*n* = 13 cells/ 10 mice) and WT (*n* = 13 cells/ 8 mice)

Given that *I*_NaL_-dependent diastolic SR Ca^2+^ release can induce an NCX mediated depolarizing current leading to cellular arrhythmias, we tested the effects of Na_V_1.8 on afterdepolarizations and spontaneous action potentials. The incidence of EADs in human atrial cardiomyocytes (3.1 ± 0.9/min, *n* = 14 cardiomyocytes/4 patients) could be significantly reduced by inhibiting Na_V_1.8 with either A-803467 (0.6 ± 0.4/min, *n *= 11/4) or PF-01247324 (0.4 ± 0.1/min, *n* = 11/4, Fig. 7a, b). Moreover, DADs and spontaneous action potentials during rest (13.6 ± 2.7/min in control, *n* = 11/4) were significantly less common when Na_V_1.8 was inhibited (A-803467: 4.5 ± 1.5/min, *n* = 9/4, PF-01247324: 5.5 ± 1.6/min, *n* = 10/4, Fig. 7c, d). Likewise, Na_V_1.8 inhibition by PF-01247324 (*n* = 13 cells/8 mice) strongly suppressed DAD occurrence and spontaneous action potentials during rest compared to control in WT (*n* = 14 cells/8 mice). Application of PF-01247324 (*n *= 13 cells/10 mice) had no effect in SCN10A^−/−^ mice, which show an already reduced incidence of arrhythmic events (*n* = 13 cells/10 mice, Fig. 7e, f). Thus, Na_V_1.8 inhibition markedly prevented cellular arrhythmias in human and murine atrial cardiomyocytes.

### SCN10A^−/−^ mice are protected against AF induction

**Fig. 8:**
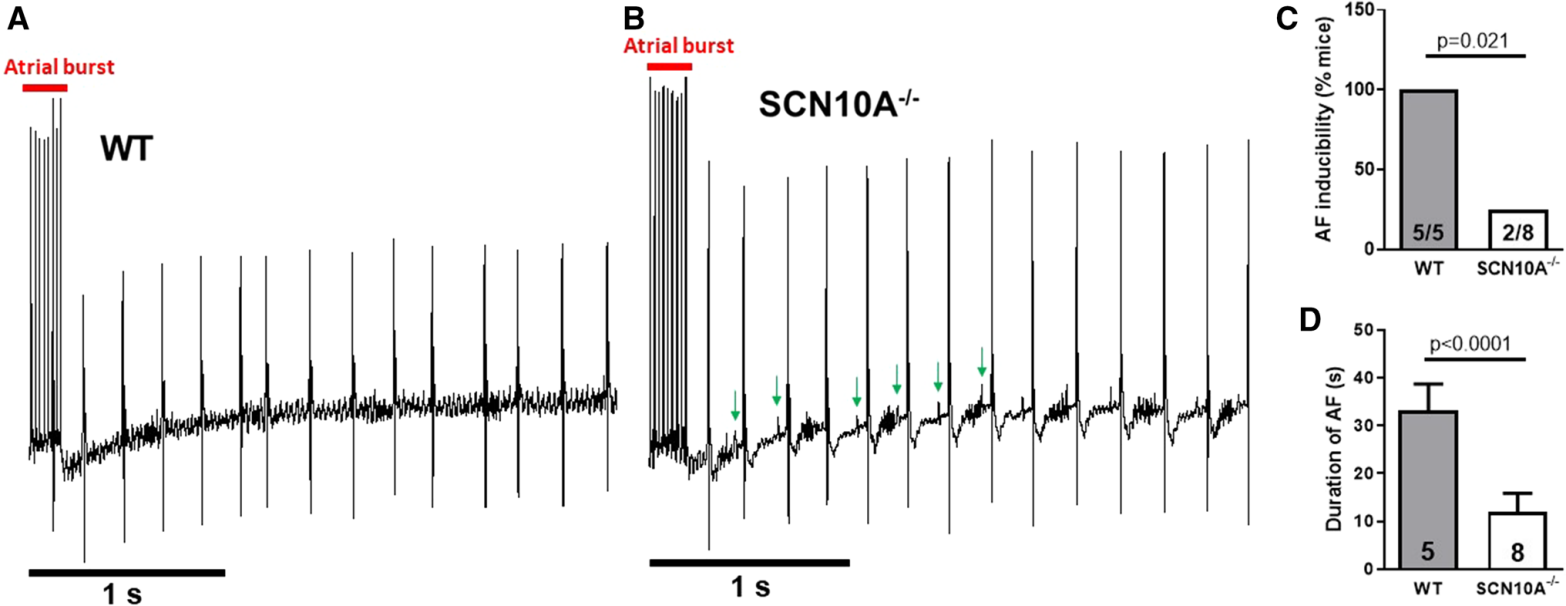
SCN10A^−/−^ mice are protected against AF induction. Data are presented as mean ± SEM. **a**, **b** Representative electrocardiogram recordings of wild type (WT) and SCN10A^−/−^ mice undergoing burst stimulation protocol. Arrows indicate regular *P* waves. **c** Percentage of inducible WT (*n* = 5) or SCN10A^−/−^ mice (*n* = 8). *P* value was calculated using Fisher's exact test. **d** AF duration after burst stimulation (5 episodes/mice) in WT (*n* = 5) and SCN10A^−/−^ mice (*n* = 8). *P* value was calculated using Mann–Whitney test

The role of Na_V_1.8 for in vivo arrhythmias was investigated using SCN10A^−/−^ mice and respective WT. After transjugular vein catheterization, five episodes of atrial burst stimulation were performed in anaesthetized mice (Fig. 8a). Electrocardiograms of SCN10A^−/−^ mice showed no changes in cardiac conduction and repolarization compared to WT (Suppl. Table 1). In WT mice undergoing atrial burst stimulation AF was inducible in all animals (*n* = 5 mice). However, only in two out of eight SCN10A^−/−^ mice AF could be induced indicating a significantly lower susceptibility to AF (*n* = 8 mice, Fig. 8b). Moreover, the AF duration after respective burst episodes was markedly shorter in SCN10A^−/−^ mice (12.0 ± 3.8 s) compared to WT (33.2 ± 5.5 s, Fig. 8c). These data demonstrate that Na_V_1.8 ablation is protective against AF induction and thereby confirm its arrhythmic potency in an in vivo system.

## Discussion

This study comprehensively investigated Na_V_1.8 in human atrial myocardium and its role in cellular electrophysiology and arrhythmogenesis. We could detect relevant Na_V_1.8 mRNA and protein levels in the human atrium. While pharmacological Na_V_1.8 modulation showed no significant effects on action potentials, it depicted a contribution to *I*_NaL_ generation and thereby to diastolic SR Ca^2+^ leak in human atrial cardiomyocytes. Importantly, selective inhibition of Na_V_1.8 with two agents potently reduced cellular arrhythmogenic triggers. These findings could be confirmed in mice lacking Na_V_1.8 (SCN10A^−/−^). Finally, in vivo studies revealed that SCN10A^−/−^ mice are protected against AF induction.

We not only found that Na_V_1.8 is expressed in the human atrium but could show that mRNA and protein expression is higher in atrial compared to ventricular myocardium. The presence of Na_V_1.8 in the human atria was indirectly suggested by genome-wide association studies (GWAS) reporting that the SCN10A gene (encoding Na_V_1.8) impacts atrial conduction, in particular PR interval and *P* wave duration [8, 18]. Data from mice further support our findings by showing a higher Na_V_1.8 expression in the atria compared to the ventricle [34]. Of note, one previous study reported a generally lower Na_V_1.8 mRNA expression in the atria compared to other Na_V_ isoforms [19] and other studies described difficulties in the detection of Na_V_1.8, which may be due to a high rate of alternate splicing [6, 9]. Recent genetic studies demonstrated an involvement of SCN10A in atrial cellular electrophysiology and could associate SCN10A variants with AF [17, 18, 25]. We therefore investigated whether Na_V_1.8 compared to Na_V_1.5 expression might be differentially regulated in patients with SR or with AF. However, we observed no differences in Na_V_1.8 protein or mRNA expression levels between SR and AF myocardium.

We therefore investigated human atrial cardiomyocytes from patients with sinus rhythm to elucidate the cellular role of Na_V_1.8 in the human atria. In patch clamp experiments, pharmacological inhibition of Na_V_1.8 did not change APA, RMP or *dv/dt* in human atrial cardiomyocytes, which could be confirmed in SCN10A^−/−^ mice. Since *dv*/*dt* is a surrogate for the fast Na^+^ influx and hence peak Na^+^ current [5], these observations suggest that the involvement of Na_V_1.8 in the peak Na^+^ current is negligible and therefore atrial conduction may not be affected. We observed a trend towards a reduced APD after Na_V_1.8 inhibition, which however did not reach statistical significance. Thus, while we could previously show a distinct APD abbreviation upon Na_V_1.8 inhibition in ventricular cardiomyocytes [10], the impact on atrial APD appears minor. However, APD is abbreviated in AF and APD shortening may not be a suitable strategy for the treatment of AF [16]. A critical issue is that many previous experimental reports on AF treatment strategies investigated permanent AF atria with a very short action potential. However, patients with permanent or long-standing AF are probably not suitable patients for a pharmacological rhythm strategy due to advanced remodeling. Since atrial APD is differentially regulated in different cardiac diseases, i.e., atrial APD is prolonged in patients with left-ventricular dysfunction [24], further patient-specific studies are needed.

We here demonstrate that pharmacological and genetic Na_V_1.8 inhibition markedly reduced *I*_NaL_ in human and murine atrial cardiomyocytes. Previous studies in animal ventricular cardiomyocytes by Yang et al. and in human ventricular cardiomyocytes by our group described a reduction of *I*_NaL_ as well as an abbreviation of APD due to Na_V_1.8 inhibition [10, 34]. SCN10A variants associated with AF were also found to modulate *I*_NaL_ after transfection in ND7/23 cells, which further strengthens findings about the role of Na_V_1.8 for *I*_NaL_ [23]. Of note, we observed a clear trend towards a further *I*_NaL_ reduction in SCN10A^−/−^ cardiomyocytes after exposure to TTX suggesting that other Na_V_ isoforms still contribute to *I*_NaL_ generation. Since *I*_NaL_ directly impacts atrial arrhythmogenesis [3, 13, 28], we consecutively evaluated whether specific Na_V_1.8 inhibition could prevent cellular arrhythmias. We have previously shown that in the human atrium *I*_NaL_-mediated Na^+^ influx can induce Ca^2+^ influx via reverse-mode NCX leading to an increased cytosolic [Ca^2+^] and an enhanced incidence of Ca^2+^ sparks [13]. In the present study, selective inhibition or ablation of Na_V_1.8 markedly suppressed SR Ca^2+^ spark frequency and the total calculated diastolic Ca^2+^ leak in atrial cardiomyocytes. Most importantly, the incidence of major diastolic Ca^2+^ release events like Ca^2+^ waves, which are considered as a proarrhythmic trigger, was significantly blunted after Na_V_1.8 inhibition/ablation. Interestingly, *I*_NaL_ inhibition by TTX showed similar antiarrhythmic effects compared to Na_V_1.8 inhibition/ablation. Thus, Na_V_1.8-dependent *I*_NaL_ inhibition alone might be sufficient enough for disrupting the vicious circle of *I*_NaL_-dependent SR Ca^2+^ leak. The electrogenic exchange of Ca^2+^ against Na^+^ via NCX can induce a transient inward current (*I*_ti_) leading to depolarization of the cell, which serves as a trigger for spontaneous action potentials [32]. In human atrial cardiomyocytes, both Na_V_1.8 blockers significantly diminished the incidence of EADs and prevented the generation of DADs and spontaneous action potentials during rest. Accordingly, SCN10A^−/−^ mice and PF-01247324-treated WT cells also showed a lower incidence of triggered activity. Cellular afterdepolarizations as well as irregular action potentials are considered as a potent underlying mechanism for triggered ectopic activity/ectopic firing, which may promote and/or maintain atrial arrhythmias [16].

To translate our cellular experimental findings in an in vivo model, we here demonstrate that SCN10A^−/−^ mice were protected against AF induction by rapid pacing and the duration of induced AF was significantly shorter in these mice. Ca^2+^ sparks and DAD-related ectopic activity have been shown to trigger ectopic beats, re-entry mechanisms [7] and may also lead to dispersion of repolarization, which further increases the susceptibility to arrhythmias/AF [31]. Accordingly, Ca^2+^ sparks and DAD-related ectopic activity could previously be linked to pacing induced AF in mice [20, 27]. Thus, our in vivo data in SCN10A^−/−^ mice may serve as a translation of our mechanistic findings into an in vivo system.

Using genetic ablation, the proarrhythmic role of Na_V_1.8 in the absence of pharmacological approaches and also our findings based on the Na_V_1.8 inhibitor PF-01247324 could be confirmed. Interestingly, few association studies in patients with early onset AF also report that SCN10A variants are associated with AF susceptibility [17, 23]. Of note, as Na_V_1.8 was discussed to modulate cardiac conduction [6, 29] the influence of SCN10A expressed in cardiac neurons/ganglia [30] may theoretically contribute to our in vivo findings. However, we demonstrate a distinct functional proarrhythmogenic role of Na_V_1.8 on human and murine cardiomyocyte level. Notably, Na_V_1.8 did not change *dv/dt* and amplitude of action potentials in atrial cardiomyocytes in our study as well as in ventricular cardiomyocytes [1, 10]. In addition, the QRS complex in the ECG was also unchanged in SCN10A^−/−^ mice. In sharp contrast, Na_V_1.5 inhibition (e.g., by flecainide) and reduction of peak Na^+^ influx causing changes in cardiac conduction can adversely affect mortality by promoting arrhythmogenic mechanisms [11].

We propose Na_V_1.8-dependent selective *I*_NaL_ reduction and prevention of atrial arrhythmogenesis to constitute a novel antiarrhythmic approach in the human, in particular for atrial arrhythmias involving focal and/or ectopic activity. Importantly, the current study investigated atrial cardiomyocytes from patients with sinus rhythm (or murine atrial cardiomyocytes) stressed with isoproterenol. From a clinical point of view, patients with permanent or long-standing AF, which are characterized by advanced structural atrial remodeling, are likely not the optimal patients for a pharmacological rhythm strategy. Therefore, we believe that atrial samples from patients at high risk for triggered/ectopic activity or paroxysmal AF may be more appropriate to investigate from a translational point of view. Nevertheless, Na_V_1.8 dysregulation might also have functional implications in long-standing AF.

Taken together, the herein presented functional evidence of Na_V_1.8 in human atrial cardiomyocytes and, most importantly, the potent antiarrhythmic effects of Nav1.8 inhibition and deletion in vitro and in vivo, could lay the foundation development towards a novel therapeutic option for atrial rhythm disorders.

## Electronic supplementary material

Below is the link to the electronic supplementary material.

Supplementary file1 (DOCX 329 kb)
