# Supplementary material for: Inhibition of NaV1.8 prevents atrial arrhythmogenesis in human and mice
Source: Basic Res Cardiol. 2020 Feb 20;115(2):20. doi: 10.1007/s00395-020-0780-8 (PMC7033079; doi:10.1007/s00395-020-0780-8)
Supplement: Supplementary file 1 — Supplementary file1 (DOCX 329 kb) [file 395_2020_780_MOESM1_ESM.docx]

**Supporting Information**

**Methods**

***Human myocardial tissue:***

All procedures were performed according to the Declaration of Helsinki and were approved by the local ethics committee. Informed consent was obtained from all patients. Human atrial myocardium from patients with sinusrhythm (SR) or from patients with atrial fibrillation (AF) was acquired from atrial resections, which were performed due to operational reasons during open heart surgery (for patient characteristics, see Table 1). After explantation, the tissue was immediately placed in 4°C cooled cardioplegic solution for transportation [2, 4].

***Human cardiomyocytes isolation:***

Atrial myocardium from patients with sinus rhythm was used for cellular experiments. Before starting isolation, human atrial tissue was cleared from fat and blood vessels, then cut into very small pieces and rinsed thoroughly. The myocardium was washed three times in Ca^2+^-free solution containing (in mmol/L): NaCl 100, glucose 20, KCl 10, KH2PO4 1,2, MgCl2 5, Taurine 50, BDM 10, and MOPS 5 (pH 7.2). The tissue was incubated at 37°C under oxygen gassing in a spinner flask filled with Ca^2+^-free solution and 0,77 mg/mL of collagenase (Worthington type 1, 370 U/mg), 0.4 mg/mL of proteinase (Sigma Type XXIV, 7.0 – 14.0 U/mg). After 45 min the supernatant was discarded and fresh Ca^2+^-free solution containing only collagenase was added. The myocytes were incubated in the solution for 5–20 min and agitated using a Pasteur pipette. Enzymatic digestion was stopped by adding BCS (2%). The supernatant containing dispersed cells was removed and centrifuged (58 g, 5 min). Storage medium was added to the remaining tissue, containing (mmol/L): Taurine 20, glutamic acid 70, KCl 30, KH2PO4 10, MgCl2 1, HEPES 10, BDM 10, Glucose 11 and bovine calve serum 2% (pH 7.4, KOH, 37°C). Remaining cardiomyocytes were liberated by agitating the tissue using a serological pipette. The supernatant was centrifuged. Cell pellets were resuspended in storage medium. Only cell solutions containing elongated cardiomyocytes with clear cross striations were selected for experiments, plated on laminin-coated recording chambers, and left to settle for 30 min. Cellular experiments were conducted at room temperature.

***Quantitative Real Time PCR (qPCR)***

Human atrial tissue or ventricular non-failing tissue were snap-frozen in liquid nitrogen and stored at -80°C. RNA was isolated by use of the SV total RNA isolation System (Promega). 100 ng RNA was reverse transcribed into cDNA using standard protocols. For qPCR, 10 µL SYBR Green PCR Master Mix (Thermo Fisher), 7 µL nuclease free water, 1 µL forward and 1µl reverse Primer and 1 µL of cDNA were mixed. Q-PCR was carried out using the iQ5 Multicolor Real-Time PCR Detection System (Bio- Rad). 40 cycles of 15 sec at 95°C followed by 1 min of 60°C were used and fluorescence was measured after each cycle. After 40 cycles melt curve analysis was performed to ensure specificity of the products. Thresholds cycles were evaluated and normalized to housekeeping genes and controls. Following primer sequences (5’-3’) were used for quantitative RT-PCR analyses: SCN10A: for: TGGCAGATGACCTGGAAGAACC; rev: CGATACGGTAGCAAGTCTTGCG (Origene, cat No HP209444, NM_006514), SCN5A: for: TACACCTTTGAGTCTCTGGTCAAG; rev: AATCACACTAAAGTCCAGCCAGTT, and GAPDH: for: GTCTCCTCTGACTTCAACAGCG; rev: ACCACCCTGTTGCTGTAGCCAA.

***Western Blots***

Human atrial tissue samples from patients with sinus rhythm (SR) and atrial fibrillation (AF) were homogenized in Tris buffer containing (mmol/L) 20 Tris-HCl, 200 NaCl, 20 NaF, 1 Na_3_VO_4_, 1 DTT, 1% Triton X-100 (pH 7.4) and protease and phosphatase inhibitor cocktails (Roche Diagnostics). Protein concentration was determined by BCA assay (Pierce Biotechnology). Denatured tissue homogenates (10 min, 70˚C in 2% β-mercaptoethanol) were separated on 7.5% SDS-polyacrylamide gels, then transferred to a nitrocellulose membrane and incubated with the following primary antibodies: mouse monoclonal anti-Na_V_1.8 (1:1000, LSBio, LS-C109037), rabbit polyclonal anti-Na_V_1.5 (1:2000, Alomone labs, ASC-005), and mouse monoclonal anti-GAPDH (1:20000, BIOTREND, BTMC-A473-9) at 4˚C overnight. Secondary antibodies included HRP-conjugated goat anti-rabbit and goat anti-mouse (1:10000, Jackson Immunoresearch, 111-035-144 and 115-035-062, respectively). The membrane was incubated with secondary antibodies for 1 hour at RT. ImmobilonTM Western Chemiluminescent HRP Substrate (Millipore) was used for the chemiluminescent detection.

***Patch-Clamp Experiments***

**I_NaL_ measurements:** Ruptured-patch whole-cell voltage-clamp was used to measure I_NaL_ in atrial cardiomyocytes (SR) with microelectrodes (2-3 MΩ). Pipettes were filled with (mmol/L) 95 CsCl, 40 Cs-glutamate, 10 NaCl, 0.92 MgCl_2_, 5 Mg-ATP, 0.3 Li-GTP, 5 HEPES, 0.03 niflumic acid (to block Ca-activated chloride current), 0.02 nifedipine (to block Ca^2+^ current), 0.004 strophanthidin (to block NCX current) 1 EGTA, and 0.36 CaCl_2_ (free [Ca^2+^]_i_,100 nmol/L) (pH 7.2, CsOH). The bath solution contained (mmol/L) 135 NaCl, 5 tetramethylammonium chloride, 4 CsCl, 2 MgCl_2_, 10 glucose, 10 HEPES (pH 7.4, CsOH). Access resistance was <7 MΩ. Cardiomyocytes were held at -120 mV and I_NaL_ was elicited using a train of pulses to -35 mV (1000 ms duration, 10 pulses, BCL 2s). Recordings were initiated 3 min after rupture. The measured current was integrated (between 100-500 ms) and normalized to the membrane capacitance.

**Action potential recordings:** For action potential recordings whole-cell patch-clamp technique was used (current clamp configuration). Microelectrodes (3-5 MΩ) were filled with (in mmol/L) 92 K-Aspartate, 48 KCl, 1 Mg-ATP, 10 HEPES, 0.02 EGTA, 0.1 GTP-Tris, and 4 Na_2_-ATP (pH 7.2, KOH). The bath solution contained (in mmol/L): 140 NaCl, 4 KCl, 1 MgCl_2_, 2 CaCl_2_, 10 Glucose, and 10 HEPES (pH 7.4, NaOH). Action potentials were continuously elicited by square current pulses of 1-2 nA amplitude and 1-5 ms duration at a frequency of 1 Hz. Access resistance was typically ~5-15 MΩ after patch rupture. Fast capacitance was compensated for in a cell-attached configuration. Membrane capacitance and series resistance were compensated after patch rupture. Signals were filtered with 2.9 and 10 kHz Bessel filters and recorded with an EPC10 amplifier (HEKA Elektronik).

***Confocal Ca***^2+^ ***spark measurements***

Isolated human atrial cardiomyocytes were loaded with a Fluo-4 AM (10 µmol/L for 15 min, Molecular Probes) at RT. The solution was substituted with Tyrode’s solution (mmol/L): NaCl 140, KCl 4, CaCl_2_ 2, MgCl_2_ 1, HEPES 5, glucose 10 (pH 7.4) and the respective agents A-803467 (30 nmol/L for 15 min, Sigma) or PF-01247324 (1 µmol/L for 15 min, Sigma). To ensure enough time for the complete de-esterification of Fluo-4 AM and drug-incubation, the cells were incubated in experimental solution for 15 min. Ca^2+^ spark measurements were obtained with a laser scanning confocal microscope (LSM 5 Pascal and LSM 7 Pascal, Zeiss) using a 40× oil-immersion objective. Fluo-4 AM was excited by an argon ion laser (488 nm), and emitted fluorescence was collected through a 505-nm long-pass emission filter. Fluorescence images were recorded in the line-scan mode with 512 pixels per line (width of each scan line: 35.57 µm) and a pixel time of 0.64 µs. One image consists of 10,000 unidirectional line scans, equating to a measurement period of 7.68 s. Experiments were conducted under resting conditions after loading the sarcoplasmic reticulum with Ca^2+^ by field stimulation at 1 Hz and 20 V. Ca^2+^ sparks were analysed with the program SparkMaster for ImageJ. The mean spark frequency of the respective cell (CaSpF) resulted from the number of sparks normalized to cell width and scan rate (100 µm/s). Cardiomyocytes exhibiting major arrhythmic events (Ca^2+^ waves, spontaneous Ca^2+^ transients, and spark clouds) were excluded from the quantification of the Ca^2+^ spark and classified as arrhythmic cells. The portion of arrhythmic cells out of all measured cells and the frequency of arrhythmic events were quantified and compared between groups.

***In-vivo arrhythmias in SCN10A^-/-^ mice***

The animal investigations conform to the “Guide for the Care and Use of Laboratory Animals” published by the US National Institutes of Health (publication No. 85-23, revised 1996) and the guidelines from Directive 2010/63/EU of the European Parliament on the protection of animals used for scientific purposes. We used ~16 week-old SCN10A^-/-^ mice [1] and compared them with their age- and sex-matched wild-type (WT) littermates. Mice were anesthetized using intraperitoneal injections of medetomidine (0.5 mg/kg), midazolam (5 mg/kg) and fentanyl (0.05 mg/kg body weight). During electrophysiological (EP) studies, body temperature was monitored by a rectal probe and controlled using a mousepad circuit board equipped with a heating element (Mousepad, THM 100, Indus Instruments, USA). All studies were performed at 37°C. We used a Millar 1.1F octapolar EP catheter (EPR-800; MillarInstruments) inserted via the right jugular vein, as previously described [3]. A computer-based data acquisition system (Powerlab 16/35; ADI Instruments) was used to record body surface ECG (lead II) and 4 intra-cardiac bipolar electrograms (Labchart Pro software, version 7; AD Instruments). QT interval was corrected for heart rate (QTc) using Mitchell’s formula. Right atrial pacing was performed using 2 ms current pulses delivered by an external stimulator (STG-3008FA; Multi Channel Systems). Atrial capture was confirmed by atrial pacing prior to the arrhythmia protocol. Mice without atrial capture were excluded from the study.

Inducibility of atrial arrhythmias was tested by decremental burst pacing. Burst pacing started at a 40 ms cycle length, decreasing by 2 ms every 2 s to a cycle length of 20 ms. Burst pacing was repeated one min after the previous burst concluded or the termination of arrhythmias. Pacing was performed 5 times in each mouse. AF was defined as the occurrence of rapid and fragmented atrial electrograms with irregular AV-nodal conduction and ventricular rhythm for at least one second. At the end of the experiments, mice were killed by cervical dislocation under anaesthesia.

**Results**


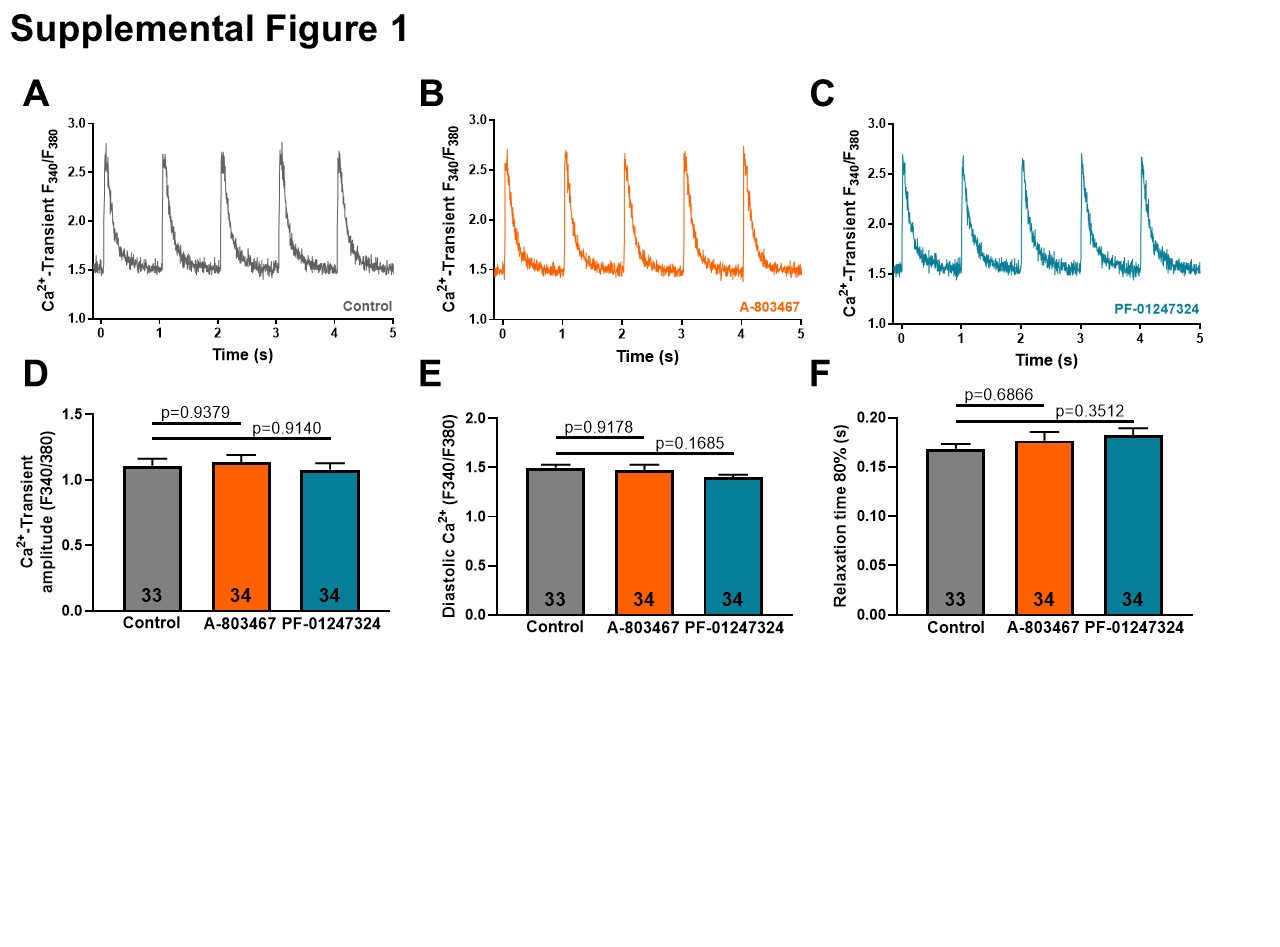


**Suppl. Fig. 1:** **(A)** Representative original recordings of stimulated systolic Ca^2+^ transients (epifluorescence microscopy, Fura-2 AM, 1Hz) of isolated murine wild-type cardiomyocytes stimulated with isoproterenol and after Na_V_1.8 inhibition by A-803467 **(B)** or PF-01247324 (**C)**. **(D)** Mean values ± SEM of systolic Ca^2+^ transient amplitude, **(E)** diastolic Ca^2+^ and **(F)** relaxation time 80% in cardiomyocytes treated with control (n=33 cardiomyocytes/4 mice), A-803467 (n=34/4) or PF-01247324 (n=34/4). P-values were calculated by using one-way ANOVA with Tukey's test for multiple comparisons.


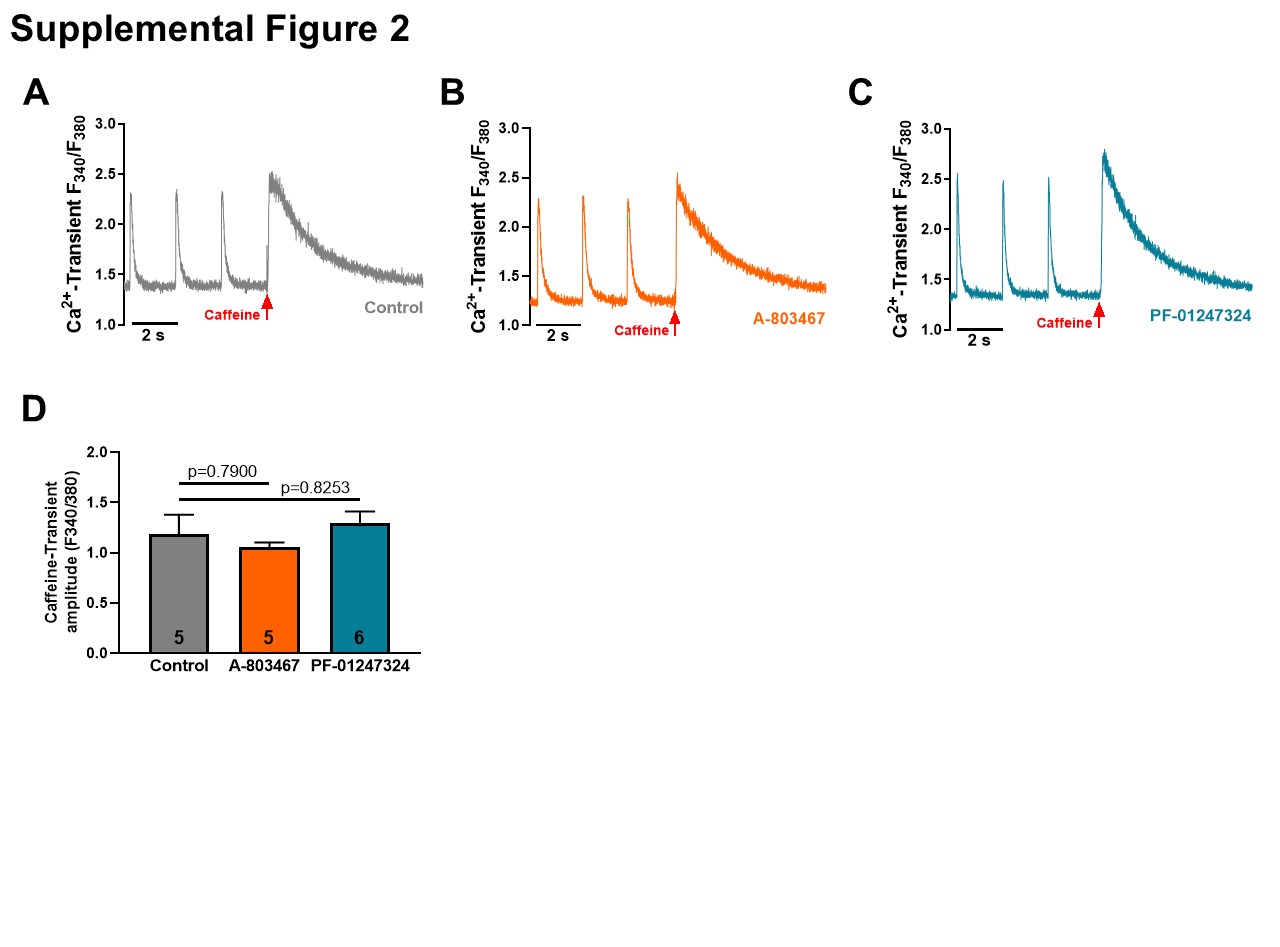


**Suppl. Fig. 2:** **(A)** Original recordings of stimulated Ca^2+^ transients and caffeine application (epifluorescence microscopy, Fura-2 AM) of isolated murine wild-type cardiomyocytes stimulated with isoproterenol and after Na_V_1.8 inhibition by A-803467 **(B)** or PF-01247324 **(C)**. **(D)** Mean values ± SEM of caffeine transient amplitude indicating sarcoplasmic Ca^2+^ load of cardiomyocytes treated with control (n=5 cardiomyocytes/4 mice), A-803467 (n=5/4) or PF-01247324 (n=6/4). P-values were computed by using one-way ANOVA with Tukey's test for multiple comparisons.


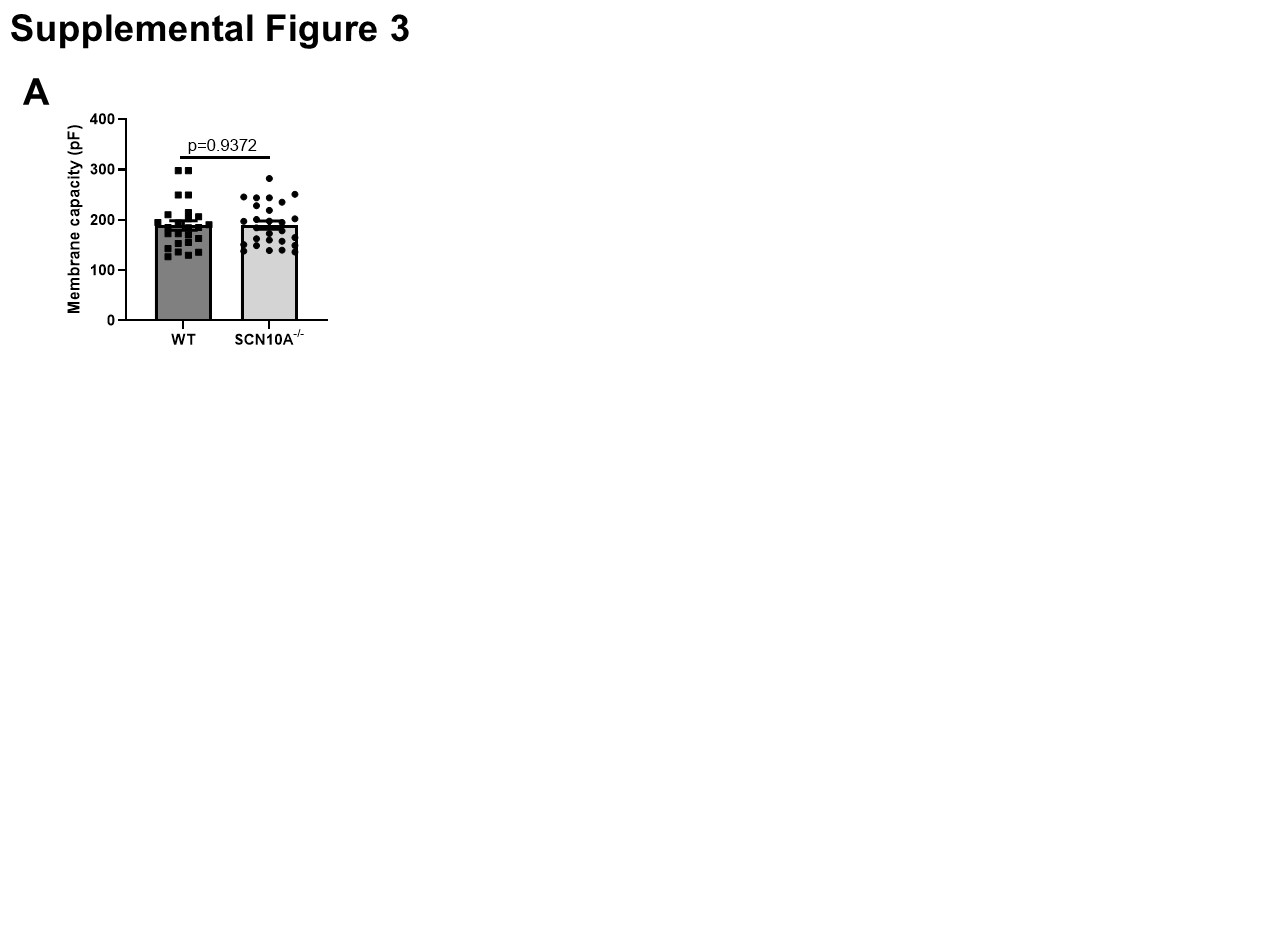


**Suppl. Fig. 3:** Membrane capacities (pF) of isolated cardiomyocytes from SCN10A^-/-^ or wild-type (WT) mice, which were used for patch clamp experiments. P-value was calculated by using student's t-test.

| **ECG Parameters** | | **WT (n=5)** | **SCN10A^-/-^ (n=8)** | **P-value** |
| --- | --- | --- | --- | --- |
| P duration (ms) | 13.2±1.7 | 15.2±2.1 | 0.5184 | |
| PR interval (ms) | | 37.1±1.5 | 39.0±1.4 | 0.3808 |
| QRS interval (ms) | | 9.9±0.3 | 9.3±0.2 | 0.1040 |
| QTc time (ms) | | 16.7±1.4 | 15.1±1.8 | 0.7780 |
| Heart rate (/min) | | 288.9±47.8 | 287.2±34.5 | 0.9777 |

**Suppl. Table 1:** Surface electrocardiogram (ECG) parameters of wildtype (WT) and SCN10A^-/-^ mice. ECG parameters were analysed in anaesthetized mice after catheterization before starting EP studies.

**References**

1. Akopian AN, Souslova V, England S, Okuse K, Ogata N, Ure J, Smith A, Kerr BJ, McMahon SB, Boyce S, Hill R, Stanfa LC, Dickenson AH, Wood JN (1999) The tetrodotoxin-resistant sodium channel SNS has a specialized function in pain pathways. Nat Neurosci 2:541-548 doi:10.1038/9195

2. Fischer TH, Herting J, Tirilomis T, Renner A, Neef S, Toischer K, Ellenberger D, Forster A, Schmitto JD, Gummert J, Schondube FA, Hasenfuss G, Maier LS, Sossalla S (2013) Ca2+/calmodulin-dependent protein kinase II and protein kinase A differentially regulate sarcoplasmic reticulum Ca2+ leak in human cardiac pathology. Circulation 128:970-981 doi:10.1161/CIRCULATIONAHA.113.001746

CIRCULATIONAHA.113.001746 [pii]

3. Purohit A, Rokita AG, Guan X, Chen B, Koval OM, Voigt N, Neef S, Sowa T, Gao Z, Luczak ED, Stefansdottir H, Behunin AC, Li N, El-Accaoui RN, Yang B, Swaminathan PD, Weiss RM, Wehrens XH, Song LS, Dobrev D, Maier LS, Anderson ME (2013) Oxidized Ca(2+)/calmodulin-dependent protein kinase II triggers atrial fibrillation. Circulation 128:1748-1757 doi:10.1161/circulationaha.113.003313

4. Sossalla S, Fluschnik N, Schotola H, Ort KR, Neef S, Schulte T, Wittkopper K, Renner A, Schmitto JD, Gummert J, El-Armouche A, Hasenfuss G, Maier LS (2010) Inhibition of elevated Ca2+/calmodulin-dependent protein kinase II improves contractility in human failing myocardium. Circ Res 107:1150-1161 doi:CIRCRESAHA.110.220418 [pii]

10.1161/CIRCRESAHA.110.220418
